# Regional-scale forest aboveground biomass mapping using temporally consistent ICESat-2, Landsat, and field inventory data

**DOI:** 10.1371/journal.pone.0330831

**Published:** 2025-09-11

**Authors:** Kasip Tiwari, Lana L. Narine, Adam Maggard, Marissa Daniel, Thomas Gallagher, Zhaofei Fan, Bikram Singh, Janaki Sandamali

**Affiliations:** 1 The Timberland Group Forestry Services Limited Liability Company, Atlanta, Georgia; 2 College of Forestry, Wildlife and Environment, Auburn University, Auburn, Alabama, United States of America; 3 Far Western University, Bhimdatta, Nepal; ICIMOD: International Centre for Integrated Mountain Development, NEPAL

## Abstract

Spatially continuous and accurate estimation of forest aboveground biomass (AGB) is essential for understanding carbon storage, ecosystem health, and biodiversity. Forests of the southeastern United States (US) represent about 40% of the nation’s forest area and one of the most significant carbon sequestration and storage potentials in the US. The availability of data from more recent and long-standing Earth-observing missions, like spaceborne light detection and ranging data from NASA’s Ice, Cloud, and land Elevation Satellite-2 (ICESat-2) and imagery from Landsat satellites, present an exemplary opportunity to characterize vegetation structure and AGB. Despite this potential, the extent to which data from these ongoing missions can be used synergistically for AGB estimation at the regional scale is not well known. This study served to better understand the combined utility of Landsat and ICESat-2 for developing a large-area AGB mapping framework. Specifically, this work served to: (1) determine the best modeling technique for estimating field-derived AGB using ICESat-2 and Landsat-derived variables, among machine learning (random forest (RF) and support vector machine (SVM)) and geostatistical approaches (random forest regression kriging (RFRK) and support vector machine regression kriging (SVMRK)), and (2) create a high-resolution (30 m) baseline AGB map for the year 2020 across ~254,266 km² of forests of the southeastern US. Canopy height information from ICESat-2, Landsat-8 imagery and imagery-derived variables, digital elevation models, and canopy cover were used to model AGB. Resulting models yielded R^2^ values ranging from 0.34 to 0.61, and RMSEs between 22 and 31 Mg/ha. Evidently, AGB estimated using the SVMRK model was substantially better than the other models (R^2^ = 0.61 and RMSE = 23.99 Mg/ha), highlighting its potential for broad-scale AGB mapping. Overall, this work highlights a feasible approach for deriving spatially comprehensive AGB information for southeastern US forests and provides a high-resolution AGB baseline product to support regional-scale monitoring.

## Introduction

Forest ecosystems play an important role in regulating global change by sequestering atmospheric carbon dioxide, thereby contributing to the mitigation of global warming [1,2]. However, carbon emissions resulting from deforestation and forest degradation negatively impact the ecosystem and the global climate [3–7]. In the southeastern United States (US), forests not only provide a consistent supply of wood and fiber but also play a significant role in carbon dynamics, sequestering about 27% of total annual carbon in the US and offsetting 13% of regional greenhouse gas emissions [8]. Therefore, the accurate quantification of forest biomass is essential and carries significant economic implications by supporting the supply of items like wood, timber, food, fiber, and energy [9,10]. Additionally, forest biomass strongly influences ecosystem sustainability, including soil and water management [11], while changes in forest biomass also impact other ecosystem services, including biodiversity [12]. The United Nations Framework Convention on Climate Change (UNFCCC), which has designated forest aboveground biomass (AGB) as an Essential Climate Variable, has highlighted the significance of forest biomass.

AGB is the sum of the weight of the portion of trees found above the ground surface when oven-dried until a constant weight is reached, typically expressed on a per-unit-area basis, i.e., Mg ha-1 or kg m-2 [13]. Around 40–50% of the plant biomass is composed of carbon, so AGB is used as a surrogate for aboveground carbon [14,15] and is important for carbon cycle studies from local to global scales [16]. Information on the spatial distribution of forest AGB is critical for estimating carbon sources and sinks [17] and mitigating greenhouse gas emissions associated with deforestation and forest degradation [18]. Accurate measurement of biomass and an analysis of its dynamics are necessary, given current concerns about global warming and ecosystem health [19,20]. Traditional methods for estimating biomass include destructive sampling and field-based inventory plots. These methods are used to estimate biomass stocks at the tree and plot level, and values are extrapolated to the studied areas with similar characteristics [21]. Although collecting field measurements is a reliable approach to estimate AGB and can be precise at a local scale, doing so across a regional scale is difficult and expensive. It is inherently limited in geographic representativeness [22–24]. Also, using only field plots is challenging for estimating AGB across a larger geographic extent due to the natural diversity in forest structure and biomass, and the rate of forest loss and disturbance [25–28].

The application of remote sensing data, calibrated and validated using field inventory information, facilitates the generation of spatially representative maps of the structure and productivity of forest ecosystems over broader regions and at lower costs [29,30]. Remote sensing is now the primary data source for broad-scale biomass estimation [24,31,32]. Forest canopy height retrieved from satellite and airborne lidar has been used to estimate biomass patterns across multiple spatial scales [33–39]. Over the past few decades, data obtained from passive sensors have played a significant role in estimating AGB [40–42]. Notably, the availability of free, medium-resolution satellite images, like Landsat, has enabled expanded usage in estimating AGB at multiple spatial scales [28,32,43–45].

Spaceborne lidar has enabled the study of AGB from local, regional, and global scales by providing three-dimensional observations or measurements of the structure of forests [46–51]. The Geoscience Laser Altimeter System (GLAS) on NASA’s Ice, Cloud, and Land Elevation Satellite (ICESat) was the first of its kind, collecting over 250 million three-dimensional observations of forest areas worldwide from 2003 to 2009 [52–54]. While GLAS was mainly designed for monitoring polar ice sheets, it was also the only spaceborne lidar system that provided three-dimensional measurements of forests. ICESat provided waveform data, which were used to estimate and map both forest canopy heights and biomass [55–58]. Following the retirement of ICESat, NASA launched the Ice, Cloud, and land Elevation Satellite-2 (ICESat-2) in 2018, and after successful completion of its nominal mission period and continued operation for over five years, it presents a valuable opportunity for modeling AGB. ICESat-2 is equipped with the Advanced Topographic Laser Altimeter System (ATLAS), which captures data at a footprint of 11 meters, delivering high-resolution details of Earth’s surface. ATLAS operates at a wavelength of 532 nm, using both strong and weak beams with an energy ratio of 4:1 [59–61]. Though primarily designed to determine changes in ice sheet elevation and mass like ICESat, it also provides a dedicated land and vegetation product (ATL08), which reports canopy height and terrain parameters at a fixed 100-meter step-size (segment) in the along-track direction. These data have been used to estimate AGB from specific site-level to broader scales [48,60,62–66]. Despite these achievements, studies focused on the estimation of AGB at a regional scale are limited, and to our knowledge, have not been reported for forests of the southeastern US.

Regional AGB estimation using spaceborne data faces several limitations, such as limited availability of ground samples, inconsistency in ground measurements, mixed pixels resulting from coarse spatial resolutions, and inconsistent pixel sizes across sample plots and satellite data [67–70]. To address these challenges and to generate full coverage estimates, three approaches are commonly used to integrate spaceborne lidar data with forest inventory information: (1) make direct linkages to field data, (2) use airborne lidar-derived information, and (3) apply extrapolated spaceborne lidar parameters as model predictors. The first approach involves geolocation-based direct linkages to field data [36,71]. For instance, spatially continuous forest biomass was generated in Northeastern China using GLAS observations and coarse-scale Moderate Resolution Imaging Spectroradiometer (MODIS) data after calibrating GLAS observations with field data spatially coincident with GLAS footprints [36]. With ICESat-2, biomass data collected from 54 ground samples were integrated with ATLAS data to estimate AGB for 74,873 footprints using a hyperparameter-optimized random forest (RF) model [64]. The second approach is to use airborne lidar data as a medium to link field-collected data with spaceborne lidar data [55,72]. For example, ATL08 data were integrated with airborne lidar and field data, along with Sentinel imagery, to extrapolate AGB over Mediterranean forests [72]. The third approach is to implement spaceborne lidar parameter extrapolation to create wall-to-wall predictors that are then applied to model AGB [47,73,74]. For instance, an ICESat-derived height parameter (RH100) was extrapolated to spatial continuous layer using a RF model and then used as a predictor for AGB estimation in China [73]. Similarly, Nandy et al. [74] retrieved the ATL08 land and vegetation product from ICESat-2 and used statistical methods to extrapolate the data to create spatially continuous layers of canopy height, instead of relating ground measurements with data at the spaceborne lidar footprint level. Authors then constructed statistical models based on ground inventory data and extrapolated ICESat-2 canopy height as one of the predictors to map AGB in the Northwest Himalayan Foothills of India. ICESat-2, like its predecessor, provides data along tracks and thus are spatially discontinuous. In addition, even if calibrated with spatially coincident field inventory data, it is challenging to create comprehensive, wall-to-wall biomass maps using this data alone. To overcome these limitations and to achieve full coverage, other spatially comprehensive information needs to be integrated using techniques capable of handling large and complex datasets.

Machine learning (ML) is a subset of computing algorithms that aims to imitate human intelligence through environmental learning. ML techniques are regarded as essential tools in the era of big data [75]. RF and support vector machine (SVM) are machine learning algorithms that have been widely applied in forest AGB estimation and mapping using remote sensing data [76–79]. Random forest regression kriging (RFRK) and support vector machine regression kriging (SVMRK) are an extension of RF and SVM, respectively, which enhances the interpolation of residuals by ordinary kriging (OK). In one study, SVM outperformed k-nearest neighbor (kNN) and RF for estimating AGB from Landsat-5 Thematic Mapper (TM) spectral reflectance data. SVM performed best following parameter optimization, otherwise, SVM could be outperformed by RF [80]. Similarly, the performance of two machine learning techniques, SVM and RF, was assessed in predicting AGB for a temperate forest of Durango state, Mexico, using Landsat 8 OLI [45]. The outcome demonstrated that the best SVM model had a root mean square error (RMSE) of 8.20 Mg/ha, which indicated good potential for AGB estimation. AGB samples in the Changbai Mountains, China, were used along with the European Space Agency’s (ESA) Sentinel imagery to develop biomass prediction models through geographically weighted regression (GWR) and ML algorithms, such as artificial neural network (ANN), SVM and RF using Sentinel datasets [81]. SVM was the best method for predicting the patterns of AGB [81]. Chen et al. [77] estimated AGB in ecoregion Changbai Mountains and eastern mountainous region of Jilin province in northeast China using multi-wavelength synthetic aperture radar (SAR) and multispectral data, and according to the findings, the random forest kriging (RFK) model outperformed the standard RF model, offering improved accuracy based on error metrics and correlation coefficients. RF/co-kriging has been found to be the most accurate and reliable method when compared with RF coupled with ordinary kriging (RF/ordinary kriging), and a RF model for AGB mapping in the subtropical forest region with complex topography of northern Guangdong province, China [82]. RF-based ordinary Kriging (RFOK) model was also used to estimate AGB for Namhton forest reserve and Yinmar forest reserve in Myanmar, producing moderate coefficients of determination values and RMSEs (R^2^: 0.47 and 0.52; RMSEs: 25 t/ha and 35t/ha), confirming their utility for AGB estimation to help determine carbon sequestration potential in the context of REDD+ [83].

The primary goal of this study was to develop a workflow for mapping AGB at the regional scale using available ICESat-2-derived and satellite imagery products. The specific objectives were to: (1) determine the best modeling technique for estimating field-derived AGB using ICESat-2 and Landsat-derived variables, among machine learning (random forest (RF) and support vector machine(SVM)) and geostatistical approaches (random forest regression kriging (RFRK) and support vector machine regression kriging (SVMRK)), and (2) create a high-resolution (30 m) baseline AGB map for the year 2020 across ~254,266 km² of forests of the southeastern US. By comparing different modelling approaches for upscaling AGB, i.e., RF, SVM, RFRK, and SVMRK, this work serves to define a framework for developing wall-to-wall AGB estimates with data from ongoing missions, to support monitoring.

## Materials and methods

### Study area

**Fig 1:**
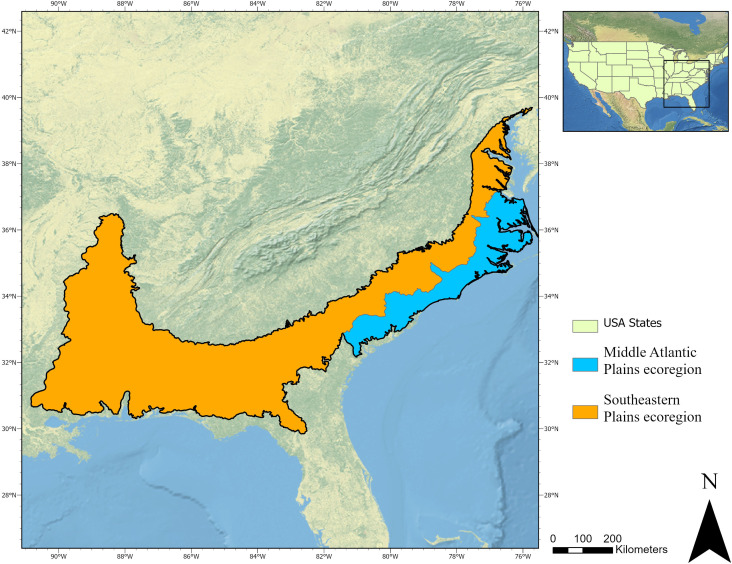
The study area in the southeastern US shows the Southeastern Plains ecoregion and the Middle Atlantic Coastal Plains ecoregion. Natural Earth Imagery is used as a basemap [88].

The study focused on two ecoregions in the southeastern US, the Southeastern Plains ecoregion and Middle Atlantic Coastal Plain ecoregion (Fig 1), representing a total forested area of 254,265.61 km^2^ [84]. The Southeastern Plains ecoregion stretches from Maryland to Mississippi, with smaller extensions into Louisiana and Tennessee, and is characterized by mild, humid subtropical climate with average annual temperatures ranging from 13°C in the north to 19°C in the south, and a mean annual precipitation of 1,358 mm. Dominant vegetation includes longleaf pine (*Pinus palustris*), loblolly pine (*Pinus taeda*), and mixed oak-hickory-pine forests, with the southern region featuring a mix of deciduous evergreens, broadleaf evergreens, and pines [85,86]. Similarly, the Middle Atlantic Coastal Plain ecoregion, extending from New Jersey to the South Carolina/Georgia line, experiences a comparable climate, with average temperatures ranging from 14°C in the north to 17°C in the south and mean annual precipitation of 1,229 mm. Forests here are dominated in the north by Shortleaf pine (*Pinus echinata*), loblolly pine, oak (Quercus spp.), cypress (*Cupressus sempervirens*), and sweetgum (*Liquidambar styraciflua*), while live oak (*Quercus virginiana*), sand laurel oak (*Quercus hemisphaerica*), and loblolly pine make up the majority of the tree species in the southern part of the ecoregion [86]. The shapefiles of the two ecoregions [87] were used to define the extent of the study area.

### Data

#### Field inventory data.

The field data were collected in the year 2020 across Arkansas, Mississippi, Florida, Alabama, Georgia, South Carolina, and North Carolina, temporally consistent with remote sensing data used for AGB mapping. A total of 14,818 geolocated plots were sampled, among them, 8,044 were variable radius plots (VRP) and 6,774 were fixed radius plots (FRP). For FRP, the diameter at breast height (DBH) of the trees, along with the per acre conversion factor, were recorded. Among 6,774 plots, 25 were 1/10th acre, 5,182 were 1/25th acre and 1,567 were 1/40th acre. Similarly, for VRP, the diameter at breast height (DBH) of the trees along with the Basal Area Factor (BAF) were recorded. Among 8,044 plots, 7,995 were of 10 BAF and 49 plots of 15 BAF. Although the field data were not originally collected with the specific aim of modelling AGB, they still offer valuable insights. Notably, 95% of these plots are located within the pine forests, which constitute approximately 55% of the total forest area in the study area [89]. This suggests a strong representation of the dominant forest type, even if the dataset may not fully capture the entire ecological variability of the area. Tree species were grouped into four primary groups: Cedar (CE), Hard hardwood (HH), Pine, and Soft hardwood (SH). AGB was calculated using the following equation [13]:


AGB = Exp(β0+β1 ln dbh)  (1)

Where,

AGB = total aboveground biomass (kg) for trees 2.5 cm dbh and larger

dbh = diameter at breast height (cm)

Exp = exponential function

ln = natural log base “e”

β0 and β1 are the constants.

β0 and β1 for the different groups [13] are:

Then, trees per acre of specific diameter in each plot was calculated [90]. For FRP, the following formulas were applied (Eqns. 2 and 3)


Trees/Acre=per acre conversion factor (2)

Whereas for VRP,


Trees/Acre=per acre conversion factor=BAF/(0.005454*DBH2) (3)

The trees per acre was multiplied by the AGB value to get AGB per acre for each tree. Then, AGB per acre for each tree in the plot were added to get total AGB per acre of the plot and used to compute AGB as Mg/ha for each plot. The AGB from the field data ranged from 1.83 Mg/ha to 278.61 Mg/ha, with a mean of 72.39 Mg/ha. The coordinates of the plots are not presented due to confidentiality requirements.

#### ICESat-2-derived canopy height.

Forest canopy height is an important predictor in the estimation of biomass [33,36,74,91]. Our earlier work with ICESat-2 involved the generation of a canopy height map for the region [92]. This map was developed for the year 2020 by upscaling the h_canopy (98th height percentile) parameter from ICESat-2’s land and vegetation product (ATL08). Mapped canopy height at a 30 m grid size with R^2^ of 0.69 and RMSE of 3.49 m was obtained by regression kriging after RF modeling [92]. This ICESat-2-derived canopy height product achieved accuracy with a Mean Absolute Error (MAE) of 2.61 and a bias of 0.02.

#### Landsat-8 data.

A total of 34 Landsat-8 images from the year 2020 with cloud cover 0–2% were downloaded from USGS Earth Explorer. Six spectral bands with a resolution of 30 m (Band 2: Blue, Band 3: Green, Band 4: Red, Band 5: Near Infrared (NIR), Band 6: Shortwave Infrared1 (SWIR1), and Band 7: Shortwave Infrared2 (SWIR2)), were extracted, and three vegetation indices (Normalized Difference Vegetation Index (NDVI) [93], Modified Soil Adjusted Vegetation Index (MSAVI) [94] and Enhanced Vegetation Index (EVI) [95]) were computed. Several studies have demonstrated utility of the selected spectral bands and vegetation indices for estimating AGB [45,96,97]. The vegetation indices were calculated as follows (Eqns. 4–6):


NDVI = (Band 5 − Band 4) / (Band 5 + Band 4) (4)

MSAVI = (2 * Band 5 + 1 − sqrt ((2 * Band 5 + 1)2 − 8 * (Band 5 − Band 4))) / 2 (5)

EVI = 2.5 * ((Band 5 − Band 4) / (Band 5 + 6 * Band 4 − 7.5 * Band 2 + 1)) (6)

The USGS Earth Resources Observation and Science (EROS) Center Science Processing Architecture (ESPA) On Demand Interface was used to download these Landsat-8-derived vegetation indices.

#### Ancillary data.

Apart from spectral (Landsat) and structural information (ICESat-2), ancillary variables listed in Table 1 were used as predictors for modeling AGB, informed by ICESat-2 vegetation studies (e.g., [48,72,92]).

**Table 1:** List of tree species with the constants β0 and β1.

Tree species	β0	β1
CE	−2.0336	2.2592
HH	−2.0127	2.4342
Pine	−2.5356	2.4349
SH	−1.9123	2.3651

### Data analysis

AGB derived from field inventory data (Section 2.2.1) was used as the dependent variable and the spectral bands and vegetation indices from Landsat imagery along with canopy height, canopy cover and DEMs (Table 2), were used as independent variables in models. ArcGIS Pro was used to mosaic extract datasets to the extent of the study area [99]. The pixel value of each predictor variable (Blue, Green, Red, NIR, SWIR1, SWIR2, NDVI, EVI, MSAVI, CC, DEM, and canopy height) was combined with the spatially coincident AGB data and the combined dataset was randomly split into 80% (n = 11,854) for model training and 20% (n = 2,964) for independent validation [100]. The machine learning models (RF and SVM), and geostatistical models (RFRK and SVMRK) were used to extrapolate AGB to the entire study area and model accuracy from each method was compared.

**Table 2:** List of ancillary variables used for estimating AGB.

Dataset	Description
Land Cover	National Land Cover Database (NLCD) provides spatially explicit data on land cover and change of the United States at intervals of two to three years between 2001 and 2021 at a 30m spatial resolution [84,98]. The NLCD 2019 land cover product was used to mask forested areas (deciduous forest, evergreen forest, mixed forest, shrub/scrub, and woody wetlands classes).
Canopy Cover (CC)	The U.S. Forest Service’s tree canopy cover product is a single percent tree canopy cover layer with values ranging from 0 to 100%. The canopy cover map (30 m spatial resolution) of NLCD 2016 was used as an independent variable for modeling.
Digital Elevation Models (DEMs)	Retrieved from USGS Earth Explorer, DEMs with a spatial resolution of one arc second (around 30 m) were resampled to match 30 m Landsat pixels and used for AGB modeling.

### AGB mapping using random forest, support vector machine and regression kriging (RFRK and SVMRK)

The RF technique developed by Breiman is a nonparametric ensemble modeling, which is resistant to overfitting, builds several tiny regression trees that contribute to predictions [101]. RF is employed as a reliable regression technique to estimate forest parameters such as biomass [48,74,102] and canopy height [103–106]. The decision tree uses a bagging or bootstrap method to generate a variety of training subsets [107,108]. The significant advantage is that several predictor variables can be added without making assumptions about their statistical distribution or covariance structure [109]. The ModelMap package in R with “model.build” function was used to generate the RF model and the “model.diagnostics” function was used to calculate the relative contributions of each predictor to the model [110]. The individual importance of the predictor variables was denoted by %IncMSE, which represents how much the model accuracy decreases when the variable is excluded.

SVM is a binary classifier for detecting outliers in regression situations with linear and nonlinear classification and intuitive model representation [24,111,112]. SVM is a supervised non-parametric statistical learning algorithm, which has demonstrated use in predicting biomass [112–115] and classifying tree species [116–119]. SVM is a well-known machine learning technique because of its stability, simplicity in tuning, and accuracy in modeling with only a few parameters [111,120]. The caret package in the R programming with “train” function was used to generate the model for SVM modeling with the argument “svmRadial”. The “varImp” function was used to calculate the relative contributions of each predictor to the model and “predict” function to obtain the final prediction map. The varImp function keeps track of how each predictor’s feature is added to the model and collects the reduction in estimate of error, for each predictor. The variable importance is measured by this overall reduction.

Regression kriging is a method for spatial prediction that combines the kriging of the residuals with the regression value of predictor variables [121,122]. The difference between reference AGB and RF/SVM estimated AGB is the known residual. Ordinary kriging (OK) was used to estimate these residuals, and the regression kriging prediction was derived by combining the kriged residuals with the RF/SVM prediction [123].


AGB(RFRK) = AGB(RF) + Rk (RF) (7)

AGB(SVMRK) = AGB(SVM) + Rk (SVM) (8)

Where AGB(RFRK) is the predicted AGB value by RFRK, AGB(RF) is the RF AGB estimates and Rk (RF) is the kriged residual for RF. Similarly, AGB(SVMRK) is the predicted AGB value by SVMRK, AGB(SVM) is the SVM AGB estimates and Rk (SVM) is the kriged residual for SVM.

OK employs a semi-variogram based on regionalized variables, to get the most unbiased estimated surface. The semi-variogram calculates the strength of the correlation between each point’s value for the studied variable and its distance from the other points [124]. Nugget, range, and sill are the three main parameters of semi-variogram. The sill represents the degree of spatial autocorrelation, while the nugget is an observation error. The bigger value of sill in comparison to nugget thus indicates a stronger spatial autocorrelation. The range parameter identifies the distance at which the spatial autocorrelation is no longer significant [125,126]. The OK interpolation is denoted by:


$$RK=\sum_{i=1}^{n}\text{wi}.\text{ri}$$ (9)

Where, Rk is the kriged residual, wi is the weight associated with the measured residuals of AGB and ri is the residual at location i. Using the geostatistical wizard in ArcGIS Pro, the OK of the residual was carried out. Then, the two raster layers of RF/SVM estimated AGB and kriged residuals were added in raster calculator to obtain the final prediction surface of AGB.

### Accuracy assessment

To assess the accuracy of the AGB estimates derived from RF, SVM, RFRK and SVMRK, statistical values were calculated based on the AGB values of test data. The statistical measures include i. The coefficient of determination (R^2^), ii. The Root Mean Square Error (RMSE), iii. The Mean Average Error (MAE), and iv. The mean bias.


$$R^{2}=1-\;\frac{\sum_{i=1}^{n}{\left(\overset{―}{y}-y_{i}\right)}^{2}}{\sum_{i=1}^{n}{\left(y_{i}-\;\overset{―}{y}\right)}^{2}}$$ (10)

$$RMSE=\sqrt{\frac{1}{n}\sum_{i=1}^{n}{\left(\overset{―}{y}-\;y_{i}\right)}^{2}}$$ (11)

$$\text{MAE}=\frac{1}{n}\sum_{i=1}^{n}\left|xi-yi\right|$$ (12)

$$\text{B}ias=\frac{1}{n}\sum_{i=1}^{n}\left(xi-yi\right)$$ (13)

Where x is the AGB estimated from RF, SVM, RFRK and SVMRK, y is the observed AGB from test data, and x’ and y’ are the average of the estimated and observed values, respectively. To measure performance improvement between the models, we calculated the relative improvement (RI) index. The RI index between RF and RFRK can be calculated using Equation 14. We can replace the RF and RFRK by SVM and SVMRK respectively in Equation 14 to calculate the RI index between SVM and SVMRK.


$$\text{RI}=\;\frac{RMSE(RF)-RMSE(RFRK)}{RMSE(RF)}$$ (14)

## Results

### AGB mapping using RF and RFRK

**Fig 2:**
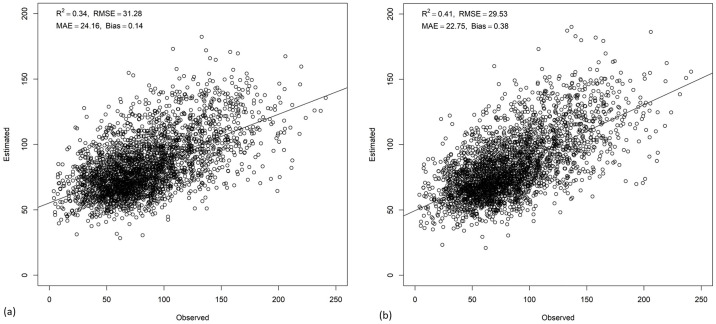
Scatterplots showing estimated versus observed AGB at a 30 m spatial resolution with test data for (a) random forest and (b) random forest regression kriging.

**Fig 3:**
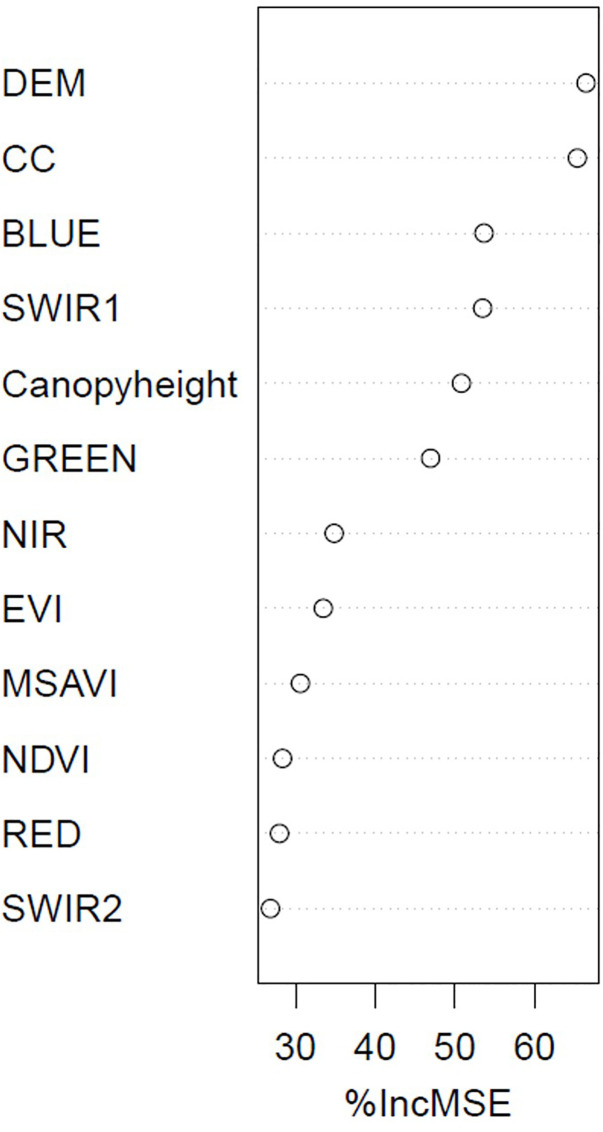
Importance of the predictor variables for estimating AGB using a random forest model. %IncMSE represents by how much the model accuracy decreases when the variable is excluded.

**Fig 4:**
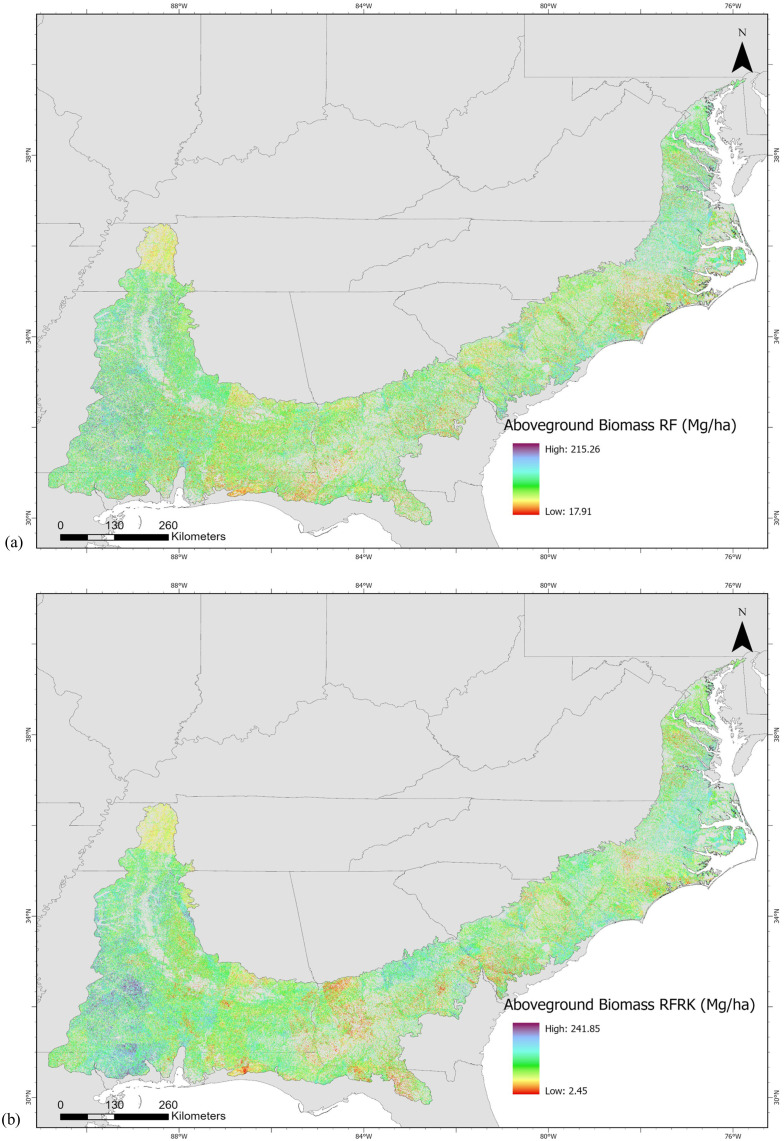
Aboveground biomass maps at 30 m spatial resolution estimated by (a) random forest and (b) random forest regression kriging. A US states shapefile is used as a basemap [127].

The resulting AGB map from the RF model ranged from 17.90 to 215.26 Mg/ha with a mean of 82.70 Mg/ha and standard deviation of 22.02 Mg/ha, whereas with the RFRK model produced a wider AGB range from 2.45 to 241.85 Mg/ha with a slightly lower mean of 81.67 Mg/ha and a higher standard deviation of 24.07 Mg/ha, indicating greater spatial variability in AGB estimation. The withheld test data (20% of data) (Fig 2) showed that the RF and RFRK produced a R^2^ value of 0.34 and 0.41, respectively. The RFRK model yielded a RMSE of 29.53 Mg/ha which is less than that of RF model’s RMSE of 31.28 Mg/ha. The MAE and bias of RFRK measured 22.75 and −0.38, respectively, while the MAE and bias of RF measured 24.16 and −0.14, respectively. It showed that the estimation error was less in the RFRK model than that of the RF model. The RI index showed that the AGB estimation of RFRK improved by 5.59% compared to that of RF. Regarding variable importance (Fig 3), the DEM contributed most to the RF model followed by CC and BLUE band. Canopy height was the fifth important predictor variable in the model. Hence, comparing different statistical measures between estimated and observed AGB, RFRK estimates AGB better than that of RF. Fig 4 shows the resulting AGB maps from RF and RFRK.

### AGB mapping using SVM and SVMRK

**Fig 5:**
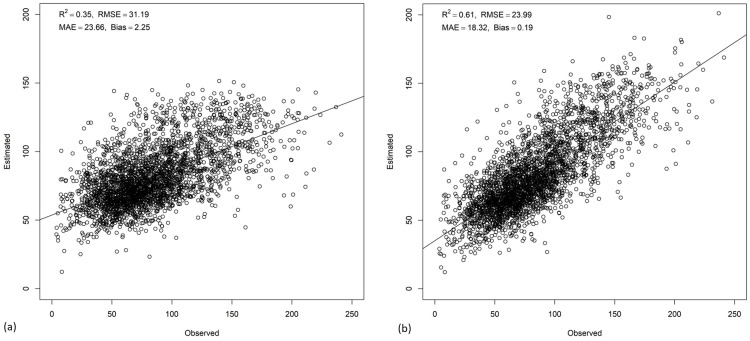
Scatterplots of estimated versus observed AGB at the 30m spatial resolution with test data for (a) support vector machine and (b) support vector machine regression kriging.

**Fig 6:**
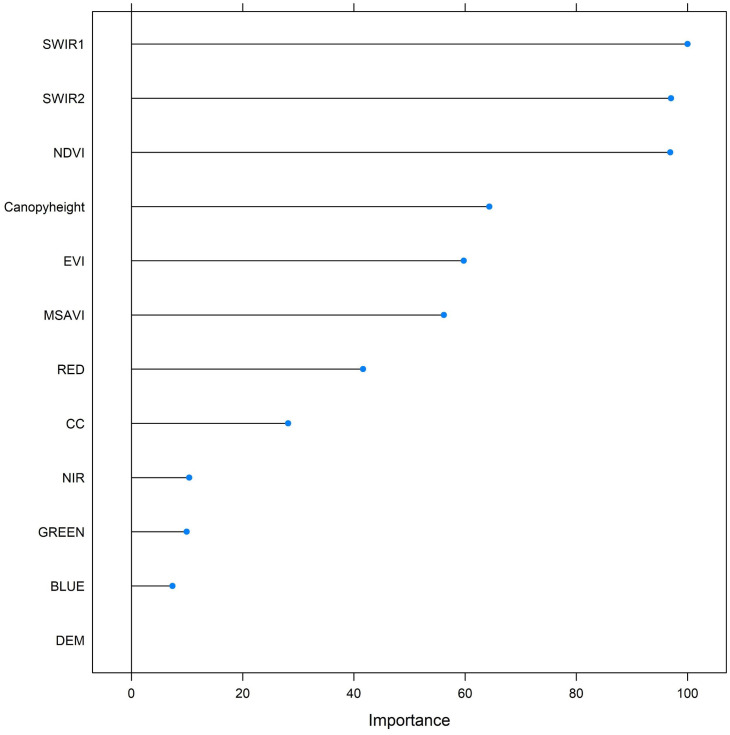
Importance of the predictor variables for estimating AGB based on support vector machine. The variable importance is measured by overall reduction in the estimate of error when added to the model.

**Fig 7:**
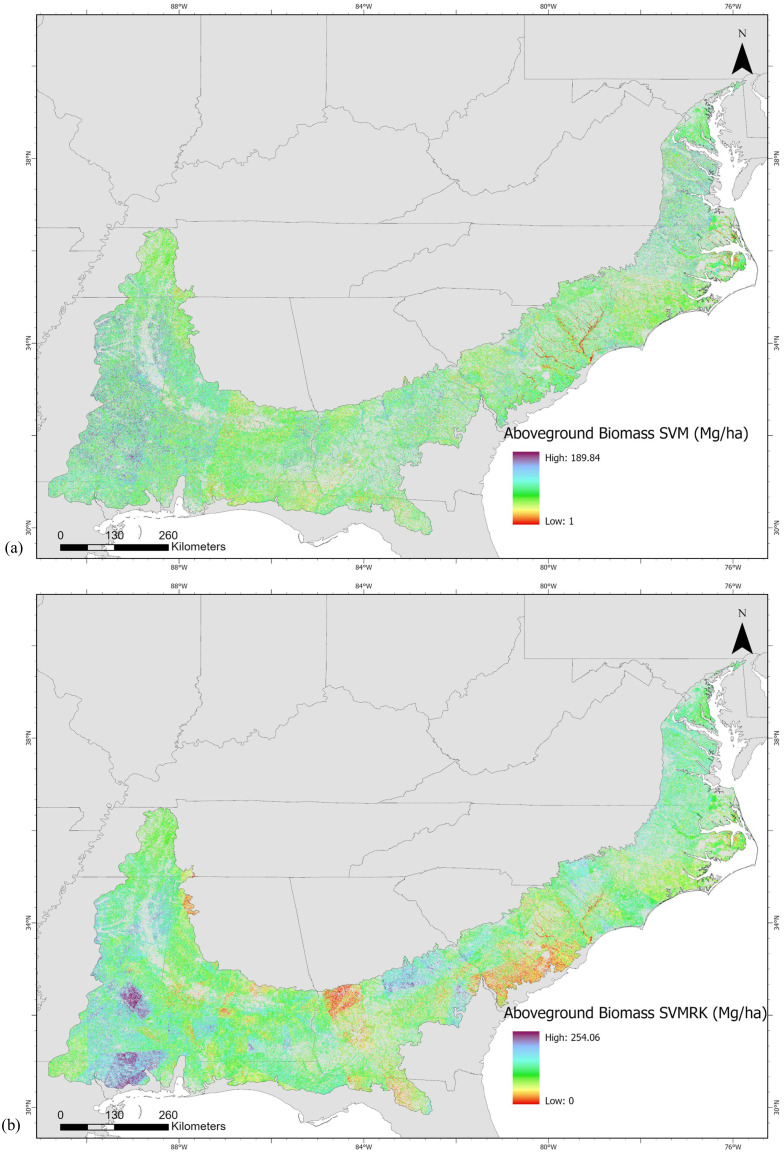
Aboveground biomass maps at 30m spatial resolution estimated by (a) support vector machine and (b) support vector machine regression kriging. US states shapefile is used as basemap [127].

AGB predictions from the SVM model ranged from 1 to 189.84 Mg/ha with a mean of 77.51 Mg/ha and standard deviation of 22.50 Mg/ha, whereas with the SVMRK model had a broader range of AGB values from 0 to 254.06 Mg/ha with a slightly higher mean of 79.32 Mg/ha and a substantially greater standard deviation of 31.68 Mg/ha indicating greater spatial variability in AGB estimation. The withheld test data (20% of data) (Fig 5) showed that the SVM and SVMRK produced a R^2^ value of 0.35 and 0.61 respectively. The SVMRK model had RMSE of 23.99 Mg/ha which is less than that of SVM model’s RMSE of 31.19 Mg/ha. Also, the MAE and bias of SVMRK were 18.32 and −0.19 respectively whereas the MAE and bias of SVM were 23.66 and −2.25 respectively. Thus, estimation errors were less in the SVMRK model than in the SVM model. The RI index showed that SVMRK improved AGB estimation by 23.08% compared to SVM. According to the SVM model (Fig 6), SWIR1 contributed most to the model followed by SWIR2 and NDVI band. The model identified canopy height as the fourth most important predictor (Fig 6). Based on the different statistical measures between estimated and observed AGB, SVMRK estimates AGB better than that of SVM. Fig 7 presents the AGB maps from SVM and SVMRK.

## Discussion

This study demonstrates the effective integration of ICESat-2derived canopy height, Landsat-8 imagery, and ancillary data with field-based AGB estimates to produce a regional AGB map at a consistent 30 m grid size across ~254,266 km^2^ in the southeastern US. Our model achieved an R^2^ of 0.61 and RMSE of 23.99 Mg/ha based on independent field validation, underscoring the potential of combining ICESat-2 and Landsat data for large-area biomass mapping.

Growing interest in AGB estimation is driven by concerns over climate change and the role of forests in carbon storage [128]. Remote sensing approaches, especially with spaceborne lidar, have expanded due to their global coverage, 3D structural data, and open-access policies [60,129]. Combining optical, radar, and lidar sensors has improved model accuracy [18,67], though regional-scale mapping still faces challenges like limited and inconsistent field data, mixed pixels from coarse resolution, and plot-imagery mismatches [67–70].

Landsat imagery, with its spectral, spatial, and temporal richness, remains a key source of AGB predictors, including vegetation indices and textural metrics [43,130–133]. Researchers consider Landsat a suitable optical data source for regional mapping of forest variables, including canopy cover, basal area, and AGB, reinforcing its value when paired with ICESat-2 for high-resolution biomass assessments [134]. ICESat-2, launched in 2018, offers enhanced resolution and vertical accuracy over its predecessor, enabling better structural detail [59–61]. While an earlier study using ICESat with MODIS enabled broader-scale AGB mapping at a coarse (500 m) resolution [135], recent efforts using ICESat-2 and Landsat have achieved 30 m AGB estimates, though mostly over small extents [48,60]. Only one prior study applied this fusion in the southern US, over a 48 km² area, reporting an R² of 0.58 and RMSE of 23.89 Mg/ha [48]. Our study addresses this gap by delivering the first large-area, high-resolution AGB map for the southeastern U.S., reinforcing the potential of ICESat-2 and Landsat integration for regional biomass monitoring.

We noted ICESat-2 canopy height as one of the important predictors in the estimation of AGB in both models examined (RF and SVM). This variable was the fifth most important predictor in the RF model and the fourth most important predictor in the SVM model of the 12 variables used. This finding is consistent with earlier work using ICESat and ICESat-2 data regarding the contribution of spaceborne lidar-derived structural information for AGB estimation. For instance, a forest AGB map for China generated by extrapolating layers from GLAS (ICESat) full-waveform parameters to use as predictor variables in a RF model produced a R^2^ of 0.75 and RMSE of 42.39 Mg/ha [73]. Similarly, Nandy et al. [74] extrapolated ICESat-2’s ATL08 canopy height parameter to generate a spatially continuous layer and then used RF to estimate and map AGB in the Northwest Himalayan Foothills of India. Authors reported a R^2^ of 0.83 and RMSE of 19.98 Mg/ha, whereas our best model estimated AGB with a R^2^ of 0.61 and RMSE of 23.99 Mg/ha. It is worth noting, however, that our study was carried out at the regional level, covering a significantly larger area of 254,256.61 km^2^. In addition, the mixed tree species of forests in the southeastern US, combined with variations in topography and climate, could contribute to differences in accuracy in estimating AGB, as compared to the above studies. In particular, Brown et al. [136] highlighted challenges with mapping AGB across heterogeneous forests within the southeastern US. Despite using airborne lidar-derived canopy structural and Sentinel-2-derived variables with linear regression and RF models, authors reported only moderate AGB prediction accuracy (R² = 0.41 and RMSE = 26.29 Mg/ha). In this study, approximately 95% of the field plots were located within pine forests, even though pine forests account for only 55% of the total forested area in the region. This disproportionate sampling likely contributed to the relatively lower model accuracy observed, as it may have biased the model toward the structural and spectral characteristics typical of pine forests, thereby limiting its ability to generalize across other forest types. The study area features heterogeneous forest structures, including mixed broadleaf forests and shrublands, all of which exhibit different structural and spectral properties. However, these were not fully represented in the training data, reducing the model’s capacity to capture the full range of forest variability, particularly when extrapolating beyond sampled plots. Nevertheless, it is important to note that our models achieved reasonable accuracies (R^2^ = 0.61) and also represent an improvement in the initial, local-scale ICESat-2 Landsat study in similar forests of the region (R^2^ = 0.58) [48]. Previous research shows that discrepancies between remote sensing spatial resolution and field plot size can significantly influence the detection of AGB variability, with greater within-pixel variability observed as the pixel-to-plot ratio increases [18,137]. Also, AGB maps often exhibit a bias at the larger scale, overestimating low AGB and underestimating high AGB values [138].

Machine learning approaches are often used to successfully generate spatially explicit estimates of forest parameters with remote sensing data combined with sample plot data. SVM has been particularly successful in applications and commended for its ability to deal with small training datasets [76]. The fact that machine learning overlooks spatial autocorrelation of nearby observed data and solely considers the relationship between the dependent variable and predictor variables is a significant flaw in the method [76,137]. Researchers use the regression kriging approach to overcome this constraint of not considering the influence of nearby data and to improve the final AGB map by minimizing variability and the high biomass saturation issue in optical remote sensing [139]. We highlight the feasibility of the SVMRK technique among the four models we used in this study, i.e., machine learning (RF and SVM) and geostatistical (RFRK and SVMRK). The AGB estimated using the SVMRK (R^2^ = 0.61 and RMSE = 23.99 Mg/ha) model was significantly better than any other model, showing SVMRK’s great potential for mapping AGB.

This work and many related studies converge on utilizing advanced modelling techniques with remote sensing data to estimate AGB in diverse forest ecosystems. In this study, both the SVMRK and RFRK models demonstrated superior performance compared to SVM and RF, which is consistent with other studies. Researchers have estimated forest AGB with a higher accuracy by applying RFRK with satellite data like ALOS-2, Sentinel-1, and Sentinel-2 [77]. Studies show RF/co-kriging consistently outperforms RF/ordinary kriging and standalone RF in complex terrain [82]. Combining RF with kriging methods and diverse remote sensing inputs improves AGB estimation by accounting for topographic and vegetation variability [83]. These findings highlight the effectiveness of combining geostatistical methods such as kriging with machine learning models to enhance the accuracy of AGB estimation in complex forest environments. Regression kriging combined with machine learning improves the accuracy of AGB estimation by lowering under- and overestimations based on remote sensing data and enhancing the capacity to monitor the forest environment [140].

Results suggest the potential of specific modelling approaches in mapping AGB at a regional scale and provide a new baseline AGB product to facilitate the development of a monitoring framework. Also, these AGB models can be utilized over various time periods to develop a time series of biomass estimates, given free and open availability of the datasets (ICESat-2 and Landsat). Future studies could investigate incorporating dynamic calibration techniques that continuously update model parameters as new field or remote sensing data become available, which may improve the model’s adaptability and accuracy, especially when applied to areas beyond the original sampling and extent. This adaptive modeling approach facilitates ongoing refinement, enabling the system to incorporate new information over time and thereby reduce uncertainties in AGB estimation across spatially or temporally variable landscapes. Although this study did not explicitly define an uncertainty estimation framework, we acknowledge its value in highlighting areas with potentially higher prediction errors. Several studies focus specifically on mapping AGB uncertainty and offer valuable direction for future work. Saarela et al. [68] applied hierarchical model-based inference to account for multiple sources of error in AGB mapping. Zhang et al. [141] applied quantile regression forests to quantify spatial uncertainty in grassland AGB estimation. Monte Carlo simulations are also employed to assess pixel-level uncertainties by integrating field, lidar, and satellite data [142]. Additionally, Johnson et al. [143] developed methods for estimating uncertainty in small area biomass averages. Integrating such approaches in future research could enhance the reliability and interpretability of AGB maps. To further strengthen model robustness and spatial generalizability, future studies should prioritize more balanced and stratified sampling across all major forest types within the region. The Global Ecosystem Dynamics Investigation (GEDI), a spaceborne lidar launched in 2018, is specifically optimized for the measurement of vegetation structure and provides datasets on canopy height, canopy cover, leaf area index, and coarse-scale (1 km) gridded AGB [129]. Both ICESat-2 and GEDI are spatially noncontiguous data sources; integrating or fusing these data may be examined to reduce the strip effect (systematic, linear patterns of bias or noise in measurements) of each individual dataset [144], improving the accuracy in mapping AGB. Similarly, ESA is recently launched the Biomass mission, in April 2025. This mission aims to advance our understanding of the role trees play in the carbon cycle and to offer critical information about the condition of our forests and how they are changing [145]. Researchers could investigate the synergistic use of these new datasets, with other full coverage products to further improve the accuracy in mapping AGB.

## Conclusion

This study is the first of its kind to use the ICESat-2 data and Landsat imagery with field inventory data to generate wall-to-wall forest AGB at the regional scale, for the southeastern US. Our findings demonstrate that researchers can develop an AGB product at a 30 m spatial resolution with these data and suggest that they use SVMRK for AGB modeling. Representing a spatially explicit baseline inventory for the year 2020, the generated AGB map supports the development of an AGB monitoring framework and products. With the ongoing availability of datasets used to derive predictors for this study (e.g., ICESat-2, Landsat), our AGB models could be applied across different time frames to generate a time series of biomass estimations.

## References

[1] Jenkins M, Schaap B. Forest Ecosystem Services. United Nations Forum on Forests; 2018.

[2] Arneth A, Harrison SP, Zaehle S, Tsigaridis K, Menon S, Bartlein PJ, et al. Terrestrial biogeochemical feedbacks in the climate system. Nature Geosci. 2010;3(8):525–32. doi: 10.1038/ngeo905

[3] Achard F, Eva HD, Mayaux P, Stibig H, Belward A. Improved estimates of net carbon emissions from land cover change in the tropics for the 1990s. Global Biogeochemical Cycles. 2004;18(2). doi: 10.1029/2003gb002142

[4] Frolking S, Palace MW, Clark DB, Chambers JQ, Shugart HH, Hurtt GC. Forest disturbance and recovery: A general review in the context of spaceborne remote sensing of impacts on aboveground biomass and canopy structure. J Geophys Res. 2009;114(G2). doi: 10.1029/2008jg000911

[5] Hansen MC, Stehman SV, Potapov PV, Loveland TR, Townshend JRG, DeFries RS, et al. Humid tropical forest clearing from 2000 to 2005 quantified by using multitemporal and multiresolution remotely sensed data. Proc Natl Acad Sci USA. 2008;105(27):9439–44. doi: 10.1073/pnas.0804042105 18591652 PMC2453739

[6] Houghton RA, Nassikas AA. Negative emissions from stopping deforestation and forest degradation, globally. Glob Chang Biol. 2018;24(1):350–9. doi: 10.1111/gcb.13876 28833909

[7] Kumar R, Kumar A, Saikia P. Deforestation and forests degradation impacts on the environment. Environmental degradation: challenges and strategies for mitigation. Springer; 2022. p. 19–46.

[8] Han FX, Plodinec MJ, Su Y, Monts DL, Li Z. Terrestrial carbon pools in southeast and south-central United States. Climatic Change. 2007;84(2):191–202. doi: 10.1007/s10584-007-9244-5

[9] De Jong W, Van Ommen JR. Biomass as a sustainable energy source for the future: fundamentals of conversion processes. John Wiley & Sons; 2014.

[10] Morris J. Recycle, Bury, or Burn Wood Waste Biomass?: LCA Answer Depends on Carbon Accounting, Emissions Controls, Displaced Fuels, and Impact Costs. J Industrial Ecology. 2016;21(4):844–56. doi: 10.1111/jiec.12469

[11] Foody GM, Boyd DS, Cutler MEJ. Predictive relations of tropical forest biomass from Landsat TM data and their transferability between regions. Remote Sensing of Environment. 2003;85(4):463–74. doi: 10.1016/s0034-4257(03)00039-7

[12] Bunker DE, Declerck F, Bradford JC, Colwell RK, Perfecto I, Phillips OL, et al. Species loss and aboveground carbon storage in a tropical forest. Science. 2005;310(5750):1029–31. doi: 10.1126/science.1117682 16239439

[13] Jenkins JC, Chojnacky DC, Heath LS, Birdsey RA. National-Scale Biomass Estimators for United States Tree Species. Forest Science. 2003;49(1):12–35. doi: 10.1093/forestscience/49.1.12

[14] Nelson R, Gobakken T, Næsset E, Gregoire TG, Ståhl G, Holm S, et al. Lidar sampling — Using an airborne profiler to estimate forest biomass in Hedmark County, Norway. Remote Sensing of Environment. 2012;123:563–78. doi: 10.1016/j.rse.2011.10.036

[15] Dinilhuda A, Akbar AA, Jumiati, Herawaty H. Potentials of mangrove ecosystem as storage of carbon for global warming mitigation. Biodiversitas. 2020;21(11). doi: 10.13057/biodiv/d211141

[16] Drake JB, Knox RG, Dubayah RO, Clark DB, Condit R, Blair JB, et al. Above‐ground biomass estimation in closed canopy Neotropical forests using lidar remote sensing: factors affecting the generality of relationships. Global Ecology and Biogeography. 2003;12(2):147–59. doi: 10.1046/j.1466-822x.2003.00010.x

[17] Chi H, Sun G, Huang J, Li R, Ren X, Ni W, et al. Estimation of Forest Aboveground Biomass in Changbai Mountain Region Using ICESat/GLAS and Landsat/TM Data. Remote Sensing. 2017;9(7):707. doi: 10.3390/rs9070707

[18] Zolkos SG, Goetz SJ, Dubayah R. A meta-analysis of terrestrial aboveground biomass estimation using lidar remote sensing. Remote Sensing of Environment. 2013;128:289–98. doi: 10.1016/j.rse.2012.10.017

[19] Le Toan T, Quegan S, Davidson MWJ, Balzter H, Paillou P, Papathanassiou K, et al. The BIOMASS mission: Mapping global forest biomass to better understand the terrestrial carbon cycle. Remote Sensing of Environment. 2011;115(11):2850–60. doi: 10.1016/j.rse.2011.03.020

[20] Li Y, Brando PM, Morton DC, Lawrence DM, Yang H, Randerson JT. Deforestation-induced climate change reduces carbon storage in remaining tropical forests. Nat Commun. 2022;13(1):1964. doi: 10.1038/s41467-022-29601-0 35413947 PMC9005651

[21] Picard N, Saint-André L, Henry M. Manual de construcción de ecuaciones alométricas para estimar el volumen y la biomasa de los árboles: del trabajo de campo a la predicción. 2012.

[22] Zianis D, Mencuccini M. On simplifying allometric analyses of forest biomass. Forest Ecology and Management. 2004;187(2–3):311–32. doi: 10.1016/j.foreco.2003.07.007

[23] Walker W, Baccini A, Nepstad M, Horning N, Knight D, Braun E. Guía de campo para la estimación de biomasa y carbono forestal. Massachusetts, USA. 2011.

[24] Lu D, Chen Q, Wang G, Liu L, Li G, Moran E. A survey of remote sensing-based aboveground biomass estimation methods in forest ecosystems. International Journal of Digital Earth. 2014;9(1):63–105. doi: 10.1080/17538947.2014.990526

[25] Asner GP, Flint Hughes R, Varga TA, Knapp DE, Kennedy-Bowdoin T. Environmental and Biotic Controls over Aboveground Biomass Throughout a Tropical Rain Forest. Ecosystems. 2008;12(2):261–78. doi: 10.1007/s10021-008-9221-5

[26] Huang W, Swatantran A, Johnson K, Duncanson L, Tang H, O’Neil Dunne J, et al. Local discrepancies in continental scale biomass maps: a case study over forested and non-forested landscapes in Maryland, USA. Carbon balance and management. 2015;10:1–16. 26294932 10.1186/s13021-015-0030-9 PMC4537504

[27] Guitet S, Hérault B, Molto Q, Brunaux O, Couteron P. Spatial Structure of Above-Ground Biomass Limits Accuracy of Carbon Mapping in Rainforest but Large Scale Forest Inventories Can Help to Overcome. PLoS One. 2015;10(9):e0138456. doi: 10.1371/journal.pone.0138456 26402522 PMC4581701

[28] López-Serrano P, López Sánchez C, Solís-Moreno R, Corral-Rivas J. Geospatial Estimation of above Ground Forest Biomass in the Sierra Madre Occidental in the State of Durango, Mexico. Forests. 2016;7(3):70. doi: 10.3390/f7030070

[29] Liang S. Recent developments in estimating land surface biogeophysical variables from optical remote sensing. Progress in Physical Geography: Earth and Environment. 2007;31(5):501–16. doi: 10.1177/0309133307084626

[30] Mcroberts R, Tomppo E. Remote sensing support for national forest inventories. Remote Sensing of Environment. 2007;110(4):412–9. doi: 10.1016/j.rse.2006.09.034

[31] Andersen H-E, Strunk J, Temesgen H, Atwood D, Winterberger K. Using multilevel remote sensing and ground data to estimate forest biomass resources in remote regions: a case study in the boreal forests of interior Alaska. Canadian Journal of Remote Sensing. 2011;37(6):596–611. doi: 10.5589/m12-003

[32] Zhu X, Liu D. Improving forest aboveground biomass estimation using seasonal Landsat NDVI time-series. ISPRS Journal of Photogrammetry and Remote Sensing. 2015;102:222–31. doi: 10.1016/j.isprsjprs.2014.08.014

[33] Lefsky MA, Harding DJ, Keller M, Cohen WB, Carabajal CC, Del Bom Espirito‐Santo F, et al. Estimates of forest canopy height and aboveground biomass using ICESat. Geophysical Research Letters. 2005;32(22). doi: 10.1029/2005gl023971

[34] Saatchi S, Marlier M, Chazdon RL, Clark DB, Russell AE. Impact of spatial variability of tropical forest structure on radar estimation of aboveground biomass. Remote Sensing of Environment. 2011;115(11):2836–49. doi: 10.1016/j.rse.2010.07.015

[35] Wang X, Ouyang S, Sun OJ, Fang J. Forest biomass patterns across northeast China are strongly shaped by forest height. Forest Ecology and Management. 2013;293:149–60. doi: 10.1016/j.foreco.2013.01.001

[36] Zhang G, Ganguly S, Nemani RR, White MA, Milesi C, Hashimoto H, et al. Estimation of forest aboveground biomass in California using canopy height and leaf area index estimated from satellite data. Remote Sensing of Environment. 2014;151:44–56. doi: 10.1016/j.rse.2014.01.025

[37] Wu X, Wang X, Wu Y, Xia X, Fang J. Forest biomass is strongly shaped by forest height across boreal to tropical forests in China. Journal of Plant Ecology. 2015:rtv001. doi: 10.1093/jpe/rtv001

[38] Yang Q, Su Y, Hu T, Jin S, Liu X, Niu C, et al. Allometry-based estimation of forest aboveground biomass combining LiDAR canopy height attributes and optical spectral indexes. Forest Ecosystems. 2022;9:100059. doi: 10.1016/j.fecs.2022.100059

[39] Musthafa M, Singh G. Forest above-ground woody biomass estimation using multi-temporal space-borne LiDAR data in a managed forest at Haldwani, India. Advances in Space Research. 2022;69(9):3245–57. doi: 10.1016/j.asr.2022.02.002

[40] Lu D. The potential and challenge of remote sensing‐based biomass estimation. International Journal of Remote Sensing. 2006;27(7):1297–328. doi: 10.1080/01431160500486732

[41] Lu D, Chen Q, Wang G, Moran E, Batistella M, Zhang M, et al. Aboveground Forest Biomass Estimation with Landsat and LiDAR Data and Uncertainty Analysis of the Estimates. International Journal of Forestry Research. 2012;2012:1–16. doi: 10.1155/2012/436537

[42] Frazier RJ, Coops NC, Wulder MA, Kennedy R. Characterization of aboveground biomass in an unmanaged boreal forest using Landsat temporal segmentation metrics. ISPRS Journal of Photogrammetry and Remote Sensing. 2014;92:137–46. doi: 10.1016/j.isprsjprs.2014.03.003

[43] Lu D. Aboveground biomass estimation using Landsat TM data in the Brazilian Amazon. International Journal of Remote Sensing. 2005;26(12):2509–25. doi: 10.1080/01431160500142145

[44] Powell SL, Cohen WB, Healey SP, Kennedy RE, Moisen GG, Pierce KB, et al. Quantification of live aboveground forest biomass dynamics with Landsat time-series and field inventory data: A comparison of empirical modeling approaches. Remote Sensing of Environment. 2010;114(5):1053–68. doi: 10.1016/j.rse.2009.12.018

[45] López-Serrano PM, Cárdenas Domínguez JL, Corral-Rivas JJ, Jiménez E, López-Sánchez CA, Vega-Nieva DJ. Modeling of Aboveground Biomass with Landsat 8 OLI and Machine Learning in Temperate Forests. Forests. 2019;11(1):11. doi: 10.3390/f11010011

[46] Hajj M, Baghdadi N, Fayad I, Vieilledent G, Bailly J-S, Minh D. Interest of Integrating Spaceborne LiDAR Data to Improve the Estimation of Biomass in High Biomass Forested Areas. Remote Sensing. 2017;9(3):213. doi: 10.3390/rs9030213

[47] Hu T, Su Y, Xue B, Liu J, Zhao X, Fang J, et al. Mapping Global Forest Aboveground Biomass with Spaceborne LiDAR, Optical Imagery, and Forest Inventory Data. Remote Sensing. 2016;8(7):565. doi: 10.3390/rs8070565

[48] Narine LL, Popescu SC, Malambo L. Using ICESat-2 to Estimate and Map Forest Aboveground Biomass: A First Example. Remote Sensing. 2020;12(11):1824. doi: 10.3390/rs12111824

[49] Jiang F, Sun H, Ma K, Fu L, Tang J. Improving aboveground biomass estimation of natural forests on the Tibetan Plateau using spaceborne LiDAR and machine learning algorithms. Ecological Indicators. 2022;143:109365. doi: 10.1016/j.ecolind.2022.109365

[50] Campbell MJ, Eastburn JF, Dennison PE, Vogeler JC, Stovall AEL. Evaluating the performance of airborne and spaceborne lidar for mapping biomass in the United States’ largest dry woodland ecosystem. Remote Sensing of Environment. 2024;308:114196. doi: 10.1016/j.rse.2024.114196

[51] Lefsky MA, Cohen WB, Parker GG, Harding DJ. Lidar remote sensing for ecosystem studies: lidar, an emerging remote sensing technology that directly measures the three-dimensional distribution of plant canopies, can accurately estimate vegetation structural attributes and should be of particular interest to forest, landscape, and global ecologists. BioScience. 2002;52(1):19–30.

[52] Wang Y, Li G, Ding J, Guo Z, Tang S, Wang C, et al. A combined GLAS and MODIS estimation of the global distribution of mean forest canopy height. Remote Sensing of Environment. 2016;174:24–43. doi: 10.1016/j.rse.2015.12.005

[53] Huang K, Pang Y, Shu Q, Fu T. Aboveground forest biomass estimation using ICESat GLAS in Yunnan, China. Yaogan Xuebao- Journal of Remote Sensing. 2013;17(1):165–79.

[54] Sun X, Abshire JB, Borsa AA, Fricker HA, Yi D, DiMarzio JP, et al. ICESat/GLAS Altimetry Measurements: Received Signal Dynamic Range and Saturation Correction. IEEE Trans Geosci Remote Sens. 2017;55(10):5440–54. doi: 10.1109/TGRS.2017.2702126 30166745 PMC6110114

[55] Boudreau J, Nelson R, Margolis H, Beaudoin A, Guindon L, Kimes D. Regional aboveground forest biomass using airborne and spaceborne LiDAR in Québec. Remote Sensing of Environment. 2008;112(10):3876–90. doi: 10.1016/j.rse.2008.06.003

[56] Zhang Y, Li W, Liang S. New Metrics and the Combinations for Estimating Forest Biomass From GLAS Data. IEEE J Sel Top Appl Earth Observations Remote Sensing. 2021;14:7830–9. doi: 10.1109/jstars.2021.3101285

[57] Wang X, Cheng X, Gong P, Huang H, Li Z, Li X. Earth science applications of ICESat/GLAS: a review. International Journal of Remote Sensing. 2011;32(23):8837–64. doi: 10.1080/01431161.2010.547533

[58] Schutz BE, Zwally HJ, Shuman CA, Hancock D, DiMarzio JP. Overview of the ICESat Mission. Geophysical Research Letters. 2005;32(21). doi: 10.1029/2005gl024009

[59] Sawruk N, Burns P, Edwards R, Litvinovitch V, Hovis F. Flight lasers transmitter development for NASA ice topography Icesat-2 space mission. In: 2018.

[60] Neuenschwander A, Pitts K. The ATL08 land and vegetation product for the ICESat-2 Mission. Remote Sensing of Environment. 2019;221:247–59. doi: 10.1016/j.rse.2018.11.005

[61] Markus T, Neumann T, Martino A, Abdalati W, Brunt K, Csatho B, et al. The Ice, Cloud, and land Elevation Satellite-2 (ICESat-2): Science requirements, concept, and implementation. Remote Sensing of Environment. 2017;190:260–73. doi: 10.1016/j.rse.2016.12.029

[62] Glenn NF, Neuenschwander A, Vierling LA, Spaete L, Li A, Shinneman DJ, et al. Landsat 8 and ICESat-2: Performance and potential synergies for quantifying dryland ecosystem vegetation cover and biomass. Remote Sensing of Environment. 2016;185:233–42. doi: 10.1016/j.rse.2016.02.039

[63] Chunyu Y, Yongchao Z, Yanqiu X, Yong P, Shiming L, Longtao C. Technical and application development study of space-borne LiDAR in forestry remote sensing. 红外与激光工程. 2020;49(11):20200235–1--10.

[64] Song H, Xi L, Shu Q, Wei Z, Qiu S. Estimate Forest Aboveground Biomass of Mountain by ICESat-2/ATLAS Data Interacting Cokriging. Forests. 2022;14(1):13. doi: 10.3390/f14010013

[65] Pang S, Li G, Jiang X, Chen Y, Lu Y, Lu D. Retrieval of forest canopy height in a mountainous region with ICESat-2 ATLAS. Forest Ecosystems. 2022;9:100046. doi: 10.1016/j.fecs.2022.100046

[66] Lin X, Xu M, Cao C, Dang Y, Bashir B, Xie B, et al. Estimates of Forest Canopy Height Using a Combination of ICESat-2/ATLAS Data and Stereo-Photogrammetry. Remote Sensing. 2020;12(21):3649. doi: 10.3390/rs12213649

[67] Ma T, Zhang C, Ji L, Zuo Z, Beckline M, Hu Y, et al. Development of forest aboveground biomass estimation, its problems and future solutions: A review. Ecological Indicators. 2024;159:111653. doi: 10.1016/j.ecolind.2024.111653

[68] Saarela S, Wästlund A, Holmström E, Mensah AA, Holm S, Nilsson M, et al. Mapping aboveground biomass and its prediction uncertainty using LiDAR and field data, accounting for tree-level allometric and LiDAR model errors. For Ecosyst. 2020;7(1). doi: 10.1186/s40663-020-00245-0

[69] Wang G, Oyana T, Zhang M, Adu-Prah S, Zeng S, Lin H, et al. Mapping and spatial uncertainty analysis of forest vegetation carbon by combining national forest inventory data and satellite images. Forest Ecology and Management. 2009;258(7):1275–83. doi: 10.1016/j.foreco.2009.06.056

[70] Wang G, Zhang M. Upscaling With Conditional Cosimulation for Mapping Above‐Ground Forest Carbon. Scale Issues in Remote Sensing. 2014:108–25.

[71] Baccini A, Goetz SJ, Walker WS, Laporte NT, Sun M, Sulla-Menashe D, et al. Estimated carbon dioxide emissions from tropical deforestation improved by carbon-density maps. Nature Clim Change. 2012;2(3):182–5. doi: 10.1038/nclimate1354

[72] Guerra-Hernández J, Narine LL, Pascual A, Gonzalez-Ferreiro E, Botequim B, Malambo L. Aboveground biomass mapping by integrating ICESat-2, SENTINEL-1, SENTINEL-2, ALOS2/PALSAR2, and topographic information in Mediterranean forests. GIScience & Remote Sensing. 2022;59(1):1509–33.

[73] Su Y, Guo Q, Xue B, Hu T, Alvarez O, Tao S, et al. Spatial distribution of forest aboveground biomass in China: Estimation through combination of spaceborne lidar, optical imagery, and forest inventory data. Remote Sensing of Environment. 2016;173:187–99. doi: 10.1016/j.rse.2015.12.002

[74] Nandy S, Srinet R, Padalia H. Mapping Forest Height and Aboveground Biomass by Integrating ICESat‐2, Sentinel‐1 and Sentinel‐2 Data Using Random Forest Algorithm in Northwest Himalayan Foothills of India. Geophysical Research Letters. 2021;48(14). doi: 10.1029/2021gl093799

[75] El Naqa I, Murphy MJ. What is machine learning? Machine learning in radiation oncology. Springer; 2015. p. 3–11.

[76] Chen L, Ren C, Zhang B, Wang Z. Multi-Sensor Prediction of Stand Volume by a Hybrid Model of Support Vector Machine for Regression Kriging. Forests. 2020;11(3):296. doi: 10.3390/f11030296

[77] Chen L, Wang Y, Ren C, Zhang B, Wang Z. Assessment of multi-wavelength SAR and multispectral instrument data for forest aboveground biomass mapping using random forest kriging. Forest Ecology and Management. 2019;447:12–25. doi: 10.1016/j.foreco.2019.05.057

[78] Sun X, Li G, Wang M, Fan Z. Analyzing the Uncertainty of Estimating Forest Aboveground Biomass Using Optical Imagery and Spaceborne LiDAR. Remote Sensing. 2019;11(6):722. doi: 10.3390/rs11060722

[79] Peng Z, Qingxun M, Jie L, Jinliang J, Ziwei L. Application of machine learning algorithms in estimation of above-ground biomass of forest. Bulletin of Surveying and Mapping. 2021;:28.

[80] López-Serrano PM, López-Sánchez CA, Álvarez-González JG, García-Gutiérrez J. A Comparison of Machine Learning Techniques Applied to Landsat-5 TM Spectral Data for Biomass Estimation. Canadian Journal of Remote Sensing. 2016;42(6):690–705. doi: 10.1080/07038992.2016.1217485

[81] Chen L, Ren C, Zhang B, Wang Z, Xi Y. Estimation of Forest Above-Ground Biomass by Geographically Weighted Regression and Machine Learning with Sentinel Imagery. Forests. 2018;9(10):582. doi: 10.3390/f9100582

[82] Su H, Shen W, Wang J, Ali A, Li M. Machine learning and geostatistical approaches for estimating aboveground biomass in Chinese subtropical forests. Forest Ecosystems. 2020;7:1–20.

[83] Wai P, Su H, Li M. Estimating Aboveground Biomass of Two Different Forest Types in Myanmar from Sentinel-2 Data with Machine Learning and Geostatistical Algorithms. Remote Sensing. 2022;14(9):2146. doi: 10.3390/rs14092146

[84] Dewitz J. National land cover database (NLCD) 2019 products. US Geological Survey. 2021;10:P9KZCM54.

[85] Griffith G. Level III North American Terrestrial Ecoregions: United States Descriptions. Prepared for the North American Commission for Environmental Cooperation; 2010:1–64.

[86] Wiken E, Nava FJ, Griffith G. North American terrestrial ecoregions—level III. Montreal, Canada: Commission for Environmental Cooperation; 2011.

[87] United States Environmental Protection Agency. Ecoregion Download Files by Region 2021 [Available from: https://www.epa.gov/eco-research/ecoregion-download-files-region

[88] NaturalEarth. 2025.

[89] USFS. Forest Type Groups of the Continental United States. ArcGIS Online. 2018.

[90] Henning JG, Mercker DC. Conducting a simple timber inventory. USA: Department of Forestry, Wildlife and Fisheries, Institute of Agriculture, University of Tennessee; 2009.

[91] Wang M, Sun R, Xiao Z. Estimation of Forest Canopy Height and Aboveground Biomass from Spaceborne LiDAR and Landsat Imageries in Maryland. Remote Sensing. 2018;10(2):344. doi: 10.3390/rs10020344

[92] Tiwari K, Narine LL. A Comparison of Machine Learning and Geostatistical Approaches for Mapping Forest Canopy Height over the Southeastern US Using ICESat-2. Remote Sensing. 2022;14(22):5651. doi: 10.3390/rs14225651

[93] Rouse J, Haas R, Schell J, Deering D, editors. Monitoring vegetation systems in the great plains with ERTS proceeding. Third Earth Reserves Technology Satellite Symposium, Greenbelt: NASA SP-351; 1974.

[94] Qi J, Chehbouni A, Huete AR, Kerr YH, Sorooshian S. A modified soil adjusted vegetation index. Remote Sensing of Environment. 1994;48(2):119–26. doi: 10.1016/0034-4257(94)90134-1

[95] Hui Qing Liu, Huete A. A feedback based modification of the NDVI to minimize canopy background and atmospheric noise. IEEE Trans Geosci Remote Sensing. 1995;33(2):457–65. doi: 10.1109/36.377946

[96] Karlson M, Ostwald M, Reese H, Sanou J, Tankoano B, Mattsson E. Mapping Tree Canopy Cover and Aboveground Biomass in Sudano-Sahelian Woodlands Using Landsat 8 and Random Forest. Remote Sensing. 2015;7(8):10017–41. doi: 10.3390/rs70810017

[97] Ahmad A, Gilani H, Ahmad SR. Forest Aboveground Biomass Estimation and Mapping through High-Resolution Optical Satellite Imagery—A Literature Review. Forests. 2021;12(7):914. doi: 10.3390/f12070914

[98] Wickham J, Stehman SV, Sorenson DG, Gass L, Dewitz JA. Thematic Accuracy Assessment of the NLCD 2016 land cover for the conterminous United States. Remote Sens Environ. 2021;257:112357. doi: 10.1016/j.rse.2021.112357 39749320 PMC11694894

[99] Esri Inc. ArcGIS Pro (Version 2.7.0) 2021 [Available from: https://www.esri.com/en-us/arcgis/products/arcgis-pro/overview

[100] Li Y, Li M, Li C, Liu Z. Forest aboveground biomass estimation using Landsat 8 and Sentinel-1A data with machine learning algorithms. Sci Rep. 2020;10(1):9952. doi: 10.1038/s41598-020-67024-3 32561836 PMC7305324

[101] Breiman L. Random Forests. Machine Learning. 2001;45(1):5–32. doi: 10.1023/a:1010933404324

[102] Mutanga O, Adam E, Cho MA. High density biomass estimation for wetland vegetation using WorldView-2 imagery and random forest regression algorithm. International Journal of Applied Earth Observation and Geoinformation. 2012;18:399–406. doi: 10.1016/j.jag.2012.03.012

[103] Fayad I, Baghdadi N, Bailly J-S, Barbier N, Gond V, Hajj M, et al. Canopy Height Estimation in French Guiana with LiDAR ICESat/GLAS Data Using Principal Component Analysis and Random Forest Regressions. Remote Sensing. 2014;6(12):11883–914. doi: 10.3390/rs61211883

[104] Ahmed OS, Franklin SE, Wulder MA, White JC. Characterizing stand-level forest canopy cover and height using Landsat time series, samples of airborne LiDAR, and the Random Forest algorithm. ISPRS Journal of Photogrammetry and Remote Sensing. 2015;101:89–101. doi: 10.1016/j.isprsjprs.2014.11.007

[105] Jin S, Su Y, Gao S, Hu T, Liu J, Guo Q. The Transferability of Random Forest in Canopy Height Estimation from Multi-Source Remote Sensing Data. Remote Sensing. 2018;10(8):1183. doi: 10.3390/rs10081183

[106] Li W, Niu Z, Shang R, Qin Y, Wang L, Chen H. High-resolution mapping of forest canopy height using machine learning by coupling ICESat-2 LiDAR with Sentinel-1, Sentinel-2 and Landsat-8 data. International Journal of Applied Earth Observation and Geoinformation. 2020;92:102163. doi: 10.1016/j.jag.2020.102163

[107] Dong J, Xiao X, Sheldon S, Biradar C, Duong ND, Hazarika M. A comparison of forest cover maps in Mainland Southeast Asia from multiple sources: PALSAR, MERIS, MODIS and FRA. Remote Sensing of Environment. 2012;127:60–73. doi: 10.1016/j.rse.2012.08.022

[108] Tian S, Zhang X, Tian J, Sun Q. Random Forest Classification of Wetland Landcovers from Multi-Sensor Data in the Arid Region of Xinjiang, China. Remote Sensing. 2016;8(11):954. doi: 10.3390/rs8110954

[109] Simard M, Pinto N, Fisher JB, Baccini A. Mapping forest canopy height globally with spaceborne lidar. J Geophys Res. 2011;116(G4). doi: 10.1029/2011jg001708

[110] Freeman EA, Frescino TS, Moisen GG. ModelMap: an R package for model creation and map production. R Package Version. 2018:4:6–12.

[111] Drake JM, Randin C, Guisan A. Modelling ecological niches with support vector machines. Journal of Applied Ecology. 2006;43(3):424–32. doi: 10.1111/j.1365-2664.2006.01141.x

[112] Deb D, Deb S, Chakraborty D, Singh JP, Singh AK, Dutta P, et al. Aboveground biomass estimation of an agro-pastoral ecology in semi-arid Bundelkhand region of India from Landsat data: a comparison of support vector machine and traditional regression models. Geocarto International. 2020;37(4):1043–58. doi: 10.1080/10106049.2020.1756461

[113] Ying G, Li Z-y, Chen E-x, He Q-s, editors. Estimation of forest biomass using Support Vector machines from comprehensive remote sensing data. 2011 International Conference on Remote Sensing, Environment and Transportation Engineering. IEEE; 2011.

[114] Guo Y, Li Z, Zhang X, Chen E-x, Bai L, Tian X, et al. editors. Optimal support vector machines for forest above-ground biomass estimation from multisource remote sensing data. 2012 IEEE International Geoscience and Remote Sensing Symposium. IEEE; 2012.

[115] Mirik M, Chaudhuri S, Surber B, Ale S, Ansley RJ. Evaluating biomass of Juniper Trees (Juniperus pinchotii) from imagery-derived canopy area using the support vector machine classifier. 2013.

[116] Heikkinen V, Tokola T, Parkkinen J, Korpela I, Jaaskelainen T. Simulated Multispectral Imagery for Tree Species Classification Using Support Vector Machines. IEEE Trans Geosci Remote Sensing. 2010;48(3):1355–64. doi: 10.1109/tgrs.2009.2032239

[117] Colgan MS, Baldeck CA, Féret J-B, Asner GP. Mapping Savanna Tree Species at Ecosystem Scales Using Support Vector Machine Classification and BRDF Correction on Airborne Hyperspectral and LiDAR Data. Remote Sensing. 2012;4(11):3462–80. doi: 10.3390/rs4113462

[118] Zhang Z, Liu X. Support vector machines for tree species identification using LiDAR-derived structure and intensity variables. Geocarto International. 2013;28(4):364–78. doi: 10.1080/10106049.2012.710653

[119] Aminuddin R, Maskan FA, Jalil UMA, Fesol SFA, Ibrahim S, editors. Support Vector Machine-based approach for Recognizing Bonsai Species using Leaf Image. 2022 IEEE 18th International Colloquium on Signal Processing & Applications (CSPA); IEEE; 2022.

[120] Adam E, Mutanga O, Odindi J, Abdel-Rahman EM. Land-use/cover classification in a heterogeneous coastal landscape using RapidEye imagery: evaluating the performance of random forest and support vector machines classifiers. International Journal of Remote Sensing. 2014;35(10):3440–58. doi: 10.1080/01431161.2014.903435

[121] Goovaerts P, editor Kriging vs stochastic simulation for risk analysis in soil contamination. geoENV I—Geostatistics for Environmental Applications: Proceedings of the Geostatistics for Environmental Applications Workshop, Lisbon, Portugal, 18–19 November 1996. Springer; 1997.

[122] Hengl T, Heuvelink GBM, Stein A. A generic framework for spatial prediction of soil variables based on regression-kriging. Geoderma. 2004;120(1–2):75–93. doi: 10.1016/j.geoderma.2003.08.018

[123] Tsui OW, Coops NC, Wulder MA, Marshall PL. Integrating airborne LiDAR and space-borne radar via multivariate kriging to estimate above-ground biomass. Remote Sensing of Environment. 2013;139:340–52. doi: 10.1016/j.rse.2013.08.012

[124] Isaaks EH, Srivastava MR. Applied geostatistics. 1989.

[125] Tang G, Yang X. ArcGIS Experimental Course for Spatial Analysis. Beijing, China: Science Press; 2013.

[126] Ou Y, Rousseau AN, Wang L, Yan B. Spatio-temporal patterns of soil organic carbon and pH in relation to environmental factors—A case study of the Black Soil Region of Northeastern China. Agriculture, Ecosystems & Environment. 2017;245:22–31. doi: 10.1016/j.agee.2017.05.003

[127] UnitedStatesCensusBureau. 2018.

[128] Kumar L, Mutanga O. Remote sensing of above-ground biomass. MDPI; 2017. p. 935.

[129] Dubayah R, Blair JB, Goetz S, Fatoyinbo L, Hansen M, Healey S, et al. The Global Ecosystem Dynamics Investigation: High-resolution laser ranging of the Earth’s forests and topography. Science of Remote Sensing. 2020;1:100002. doi: 10.1016/j.srs.2020.100002

[130] Coops NC, Tompalski P, Goodbody TRH, Queinnec M, Luther JE, Bolton DK, et al. Modelling lidar-derived estimates of forest attributes over space and time: A review of approaches and future trends. Remote Sensing of Environment. 2021;260:112477. doi: 10.1016/j.rse.2021.112477

[131] Du H, Zhou G, Ge H, Fan W, Xu X, Fan W, et al. Satellite-based carbon stock estimation for bamboo forest with a non-linear partial least square regression technique. International Journal of Remote Sensing. 2011;33(6):1917–33. doi: 10.1080/01431161.2011.603379

[132] Maake R, Mutanga O, Chirima G, Sibanda M. Quantifying aboveground grass biomass using space-borne sensors: A meta-analysis and systematic review. Geomatics. 2023;3(4):478–500.

[133] Zheng D, Rademacher J, Chen J, Crow T, Bresee M, Le Moine J, et al. Estimating aboveground biomass using Landsat 7 ETM+ data across a managed landscape in northern Wisconsin, USA. Remote Sensing of Environment. 2004;93(3):402–11. doi: 10.1016/j.rse.2004.08.008

[134] Matasci G, Hermosilla T, Wulder MA, White JC, Coops NC, Hobart GW, et al. Large-area mapping of Canadian boreal forest cover, height, biomass and other structural attributes using Landsat composites and lidar plots. Remote Sensing of Environment. 2018;209:90–106. doi: 10.1016/j.rse.2017.12.020

[135] Chi H, Sun G, Huang J, Guo Z, Ni W, Fu A. National Forest Aboveground Biomass Mapping from ICESat/GLAS Data and MODIS Imagery in China. Remote Sensing. 2015;7(5):5534–64. doi: 10.3390/rs70505534

[136] Brown S, Narine LL, Gilbert J. Using Airborne Lidar, Multispectral Imagery, and Field Inventory Data to Estimate Basal Area, Volume, and Aboveground Biomass in Heterogeneous Mixed Species Forests: A Case Study in Southern Alabama. Remote Sensing. 2022;14(11):2708. doi: 10.3390/rs14112708

[137] Guo P-T, Li M-F, Luo W, Tang Q-F, Liu Z-W, Lin Z-M. Digital mapping of soil organic matter for rubber plantation at regional scale: An application of random forest plus residuals kriging approach. Geoderma. 2015;237–238:49–59. doi: 10.1016/j.geoderma.2014.08.009

[138] Avitabile V, Herold M, Heuvelink GBM, Lewis SL, Phillips OL, Asner GP, et al. An integrated pan-tropical biomass map using multiple reference datasets. Glob Chang Biol. 2016;22(4):1406–20. doi: 10.1111/gcb.13139 26499288

[139] Silveira EMO, Espírito Santo FD, Wulder MA, Acerbi Júnior FW, Carvalho MC, Mello CR, et al. Pre-stratified modelling plus residuals kriging reduces the uncertainty of aboveground biomass estimation and spatial distribution in heterogeneous savannas and forest environments. Forest Ecology and Management. 2019;445:96–109. doi: 10.1016/j.foreco.2019.05.016

[140] Li Y, Li M, Liu Z, Li C. Combining Kriging Interpolation to Improve the Accuracy of Forest Aboveground Biomass Estimation Using Remote Sensing Data. IEEE Access. 2020;8:128124–39. doi: 10.1109/access.2020.3008686

[141] Zhang S, Wu T, Gao P, Liu Y. Uncertainty assessment of grassland aboveground biomass using quantile regression forests. J Appl Rem Sens. 2024;18(04). doi: 10.1117/1.jrs.18.044507

[142] Urbazaev M, Thiel C, Cremer F, Dubayah R, Migliavacca M, Reichstein M, et al. Estimation of forest aboveground biomass and uncertainties by integration of field measurements, airborne LiDAR, and SAR and optical satellite data in Mexico. Carbon balance and management. 2018;13:1–20. 29468474 10.1186/s13021-018-0093-5 PMC5821638

[143] Johnson LK, Domke GM, Stehman SV, Mahoney MJ, Beier CM. From pixels to parcels: flexible, practical small-area uncertainty estimation for spatial averages obtained from aboveground biomass maps. arXiv preprint arXiv:241216403. 2024.

[144] Liu X, Su Y, Hu T, Yang Q, Liu B, Deng Y, et al. Neural network guided interpolation for mapping canopy height of China’s forests by integrating GEDI and ICESat-2 data. Remote Sensing of Environment. 2022;269:112844. doi: 10.1016/j.rse.2021.112844

[145] ESA. BIOMASS. 2022.

